# Abortion Law and Human Rights in Poland: The Closing of the Jurisprudential Horizon

**DOI:** 10.1007/s40803-022-00167-9

**Published:** 2022-02-16

**Authors:** Marta Bucholc

**Affiliations:** 1 Faculty of Sociology, University of Warsaw, Karowa 18, 00-927 Warsaw, Poland; 2 Centre de Recherche en Science Politique, Université Saint-Louis Bruxelles, 119 Rue du Marais, 1000 Brussels, Belgium

**Keywords:** Poland, Abortion, Human rights, Rule of law, Constitutional law

## Abstract

On 22 October 2020, the Constitutional Tribunal of Poland ruled that an abortion due to foetal impairment was unconstitutional. This article discusses the context of this controversial ruling as well as its main tenets, focusing on the interpretation of the human rights proffered by the Tribunal and on the rule of law concerns raised by the Tribunal’s decision. Against the backdrop of a brief history of the legal regulation of abortion in Poland since 1945, the article offers a critical assessment of the human rights framework used in the Polish abortion debate. Based on a close reading of the Tribunal’s ruling and the dissenting opinions, the article points out the particularities in the Tribunal’s engagement with international law and human rights jurisprudence. The article argues that the Tribunal’s decision is yet another symptom of the crisis in which the rule of law in Poland has found itself since 2015. It bears evidence to the closing of the jurisprudential horizon caused by the political change which has been taking place in Poland since 2015, consisting of the reduction of the role of international human rights debates as a reference in Polish constitutional jurisprudence. The ruling is therefore a portent of Poland’s future compliance with its international commitments in human rights matters.

Human rights serve to protect the basic values of what may yet become the global community of humankind. That these values need careful weighing and in-depth, context-sensitive understanding is evident. That the result of the weighing of values may differ depending on where, when and by whom this is done is equally obvious: human rights depend on their local legal and cultural embedding not just for their validity and enforceability, but also for the crystallisation of their meaning in each society. Human rights have been argued to be the last universalist utopia of mankind (Moyn 2012). But they are more than that: they are a potentially powerful instrument for fostering international cooperation by asserting common humanity under a common legal standard of protection. However, what is posited as a common standard is in fact very far from that, and there are few better illustrations of the internal differentiation of the global regime of the protection of human rights than the human rights treatment of abortion (Rebouché 2016; Zampas and Gher 2008).

The regulation of abortion is a point where religious, moral, political and legal considerations converge and cross to create a global normative landscape of unsurpassed complexity. Recent decades have seen an increase in regulatory interventions in pregnancy and abortion worldwide (see United Nations 2014). While some nations have liberalized their abortion laws, others have introduced stricter prohibitions, and any simple explanation for these divergences invariably fails. It is noteworthy that the countries sharing the same overall cultural set-up that is determined, among other things, by the role of traditional religions, frequently end up having fundamentally different abortion laws. The examples include Spain and Malta in Europe, or Argentina and Honduras in South America. However, what is probably even more puzzling from a human rights perspective are the differences between the legal discourses in the countries which belong to the same regional systems for the protection of human rights and recognize the same case law in matters of pregnancy and abortion. The protection of human rights can be raised as an argument in support of radically different variants of the legal regulation of abortion, which has been argued to lead to a “global legal indeterminism” in this field (see Bunikowski 2019, p. 106). Any shifting of weight between various uses of human rights can be a reliable indicator of the turn that the legal culture of a particular society is taking within the broad framework of international law.

In this article, I will examine the signs of one such legal-cultural turn which has occurred in Poland in October 2020. Since 1993, with a short interval in 1997, abortion law in Poland has been one of the strictest in Europe, only allowing abortion in three cases: severe foetal impairment, a danger to the mother’s life or health, and pregnancy resulting from a criminal act. What was usually—rather misleadingly—dubbed the “1993 abortion compromise” has been contested unsuccessfully by various social and political forces for almost three decades. This compromise was finally abolished by a ruling of the Constitutional Tribunal on 22 October 2020 (K 1/20) that declared abortion for foetal impairment unconstitutional, a decision which has been described as a “tragic judgment” (Łętowska 2020). From the point of view of a socio-legal scholar, there is a double tragedy playing out here. On the one hand, there is a social tragedy which is always, inevitably, related to a very narrow limitation of access to legal and safe abortion. The ruling of the Constitutional Tribunal reduced the conditions under which an abortion is legal in Poland: it is not a legal prohibition on abortion, but very nearly so. On the other hand, however, there is also a dimension of the tragedy which is related to the way in which the Tribunal’s decision was argued and justified. The latter dimension of this tragedy consists of what I call the closing of the jurisprudential horizon.

My analysis begins with an introduction to the concept of the jurisprudential horizon, followed by a discussion of the intrinsic connection between human rights and the rule of law. Subsequently, I present a brief history of the legal regulation of abortion since 1945 and the human rights framing of the abortion debate in Poland in order to provide the context for an analysis of the Constitutional Tribunal’s abortion ruling of October 2020. My goal is to identify the main contentious points regarding the Tribunal’s decision as far as the interpretation of human rights is concerned. I argue that the Tribunal’s reasoning is not only in itself yet another symptom of the crisis in which the rule of law in Poland has found itself since 2015, but that it has also proved to be a portent of Poland’s further compliance with its international commitments in matters of human rights. The decision of the Polish Constitutional Tribunal of 24 November 2021 (K 6/21) that declared Art. 6 of the European Convention on Human Rights to be unconstitutional is emblematic for this process. However, the abortion ruling of October 2020, though less conspicuously engaging with the international human rights framework, is even more informative as far as the mechanism of severing the ties with international law is concerned. Instead of expressly eliminating the norms of international law by declaring them unconstitutional, the abortion ruling produces the same effect by the selection of jurisprudential arguments—a method that is just as efficient, though far less spectacular politically.

## The Concept of the Jurisprudential Horizon

The choice of sources from which the jurisprudence draws its interpretations, arguments, and justifications belongs to the foundational elements of a legal culture, just as much as the institutional context as well as the styling and phrasing of its expressions (Wieacker and Bodenheimer 1990, pp. 1–29). In this article, I use the notion of the jurisprudential horizon not just to assert the obvious: that jurisprudence is always culturally embedded, but also to convey that it is possible to trace the dynamics of broadening or narrowing down what counts as an eligible reference in jurisprudential practice. The horizon is a mobile line—it follows the movements of the perceiving subject. That is the advantage of the concept of the jurisprudential horizon over the more static idea of the legal or jurisprudential landscape, which, from time to time, has been used to describe the general characteristics of the legal system in which the legal subjects move around similar to their movements in the physical environment. The horizon is also an apparent line—there is nothing in reality which would correspond to it; it is a construct of our cognitive activity. However, its perception has very real consequences, as reflected by our use of the metaphor of broadening or narrowing down the intellectual horizons as a result of travelling, be it in body or in mind. A jurisprudence with broadening horizons would be open to new arguments and new categories of arguments, and a jurisprudence whose horizons are narrowing down would end upby excluding the arguments which had been previously considered usable and valid.

Thus, an institutional or political change may bring about a broadening of the horizon of jurisprudence. The emergence of the international regime for the protection of human rights is an example of such a process, through which international law and case law have entered the domain of national jurisprudential practice as eligible argumentative references to new sources of interpretation patterns, legitimation, and justification. But it is equally true that an institutional or political change may lead to a shrinking of the horizon of valid references by eliminating some of them. This need not occur by way of a modification of the rules by which jurisprudential practice operates, it may be a de facto change, an act of the “politics of jurisprudence” (Cotterrell 1989). For example, a radical top-down secularization may lead to the exclusion of religious arguments from the jurisprudence, thereby narrowing the jurisprudential horizon. But the same effect may be produced by a new *Zeitgeist* resulting in changing the cultural dispositions of the members of the judiciary, allowing for more religious thinking where there was little before, or the other way round. Finally, the broadening and narrowing can run parallel to each other: in fact, the shift in eligible methods of argumentation can be expected to combine an elimination of some arguments with an inclusion of others. I employ the terms “broad” and “narrow” in a purely descriptive, formal sense, to refer to the inclusiveness or exclusivity of the scope of eligible jurisprudential arguments, notwithstanding their substance. The ultimate effect of broadening or narrowing the jurisprudential horizon would, however, ultimately depend on what is being included or excluded as an eligible argument, and what are the social and cultural characteristics as well as the political circumstances of this process. As opposed to intellectual horizons, the broadening of jurisprudential horizons is not always a good thing, and keeping them narrow is not always a bad one.

I argue that the 2020 abortion ruling by the Polish Constitutional Tribunal is symptomatic of the narrowing of the jurisprudential horizon which I venture to describe as its “closing down”. This process has been instigated by a political change which has been taking place in Poland since 2015, consisting of the reduction of the role of international law and the jurisprudence on human rights as a reference in Polish constitutional jurisprudence.

## Abortion in the Global System of Human Rights

The regulation of abortion in the system of human rights is a function of its position at the intersection of various rights. The human rights which form the basic reference framework for abortion law are the right to life, the right to be free from torture and ill-treatment, the right to health, the right to privacy and bodily integrity, the right to education, and the prohibition of discrimination. A consequence of the right to health is the concept of reproductive rights, which would be protected as a special part of health referring to sexuality and sexual reproduction. The concept of reproductive rights has been recognized in UN practice since the 1990s (Metwally et al. 2019). While it is not mentioned in the UN Covenant or in most of the regional conventions, it has a treaty basis in the African regional system for the protection of human rights (Art. 14 of the Maputo Protocol of 2003). It has also been in use since the 1990s by the UN organisations, including the World Health Organisation and the United Nations Population Fund, and since then it has developed into a part of a conceptual network including not only reproductive and sexual health and sexual rights, but also reproductive justice (Zakiya and Luker 2013).^1^ The United Nations Committee on the Elimination of Discrimination against Women (CEDAW) has repeatedly expressed the view that access to safe and legal abortion, as well as related services and information, are essential aspects of women’s reproductive health, and hence they are included in human rights protection (Cook 1991). The reference to human rights has also been an important instrument in the repertoire of the movements for reproductive justice (Luna 2020).

In 2016, the United Nations Human Rights Committee found that the prohibition of torture and other forms of ill-treatment contained in Article 7 of the Covenant allows for a right to abortion in circumstances of fatal foetal abnormality (*Mellet v. Ireland* 2021, confirmed in *Whelan v. Ireland* 2021). The Committee’s decision was a milestone in the development of the global regime of reproductive rights. The common denominator of the previous jurisprudence of various adjudicative bodies was to stress the inadmissibility of the arbitrary denial of access to legal abortion as a potential violation of women’s rights to freedom from cruel, inhuman or degrading treatment and to privacy. In *Mellet*, the Human Rights Committee went a step further, addressing the prohibition of abortion as such and not the de facto difficulty in accessing legal abortion.

General Comment No. 36 of the United Nations Human Rights Committee of 3 September 2019 on the right to life (Art. 6 of the International Covenant on Civil and Political Rights) states that “the right to life is the supreme right from which no derogation is permitted” and which “should not be interpreted narrowly” (UN Human Rights Committee 2019, p. 1). The same document introduces the obligation of the parties to the Covenant to “provide safe, legal and effective access to abortion where the life and health of the pregnant woman or girl is at risk, or where carrying a pregnancy to term would cause the pregnant woman or girl substantial pain or suffering, most notably where the pregnancy is the result of rape or incest or where the pregnancy is not viable”. It also prohibits the states parties from regulating “pregnancy or abortion in all other cases in a manner that runs contrary to their duty to ensure that women and girls do not have to resort to unsafe abortions” (UN Human Rights Committee 2019, Sec. 8). This line of thinking by the UN Human Rights Committee was not, however, uniformly followed by all the UN member states and regional systems. The status of the Committee’s views expressed in its comments is weaker than that of the decisions of international courts of human rights. According to the report by the Venice Commission (2014, Sec. 78):

Because the views are not legally binding judgments, the HRC does not possess an explicit competence to ensure their enforcement. However, the authority to monitor the effect of its views is an implied power of the HRC. (…) The legal consequence is that member states are under the obligation to take the HRC’s final views into consideration in good faith.

Since the establishment of the UN in 1945, the legal governance of abortion has developed globally to include a consistent scale of possible solutions, which indicates a certain consensus in the typification of the circumstances under which an abortion may be legal. The catalogue which is used both by the United Nations (2014) and in the scholarship ranges from the most restrictive variant, in which abortion is only legal to save a woman’s life or health. Next on the scale are rape or incest, foetal impairment, then economic and social reasons, up to the most liberal variant of abortion being accessible on request, with various limitations as to the gestational periods (Guillaume and Rossier 2018). The global variety of abortion laws thus displays a clear pattern.

While abortion law is also situated at the intersection of various rights in regional systems for the promotion and protection of human rights, the balance of the rights in question differs significantly depending on the system in question. The African system has expressly recognized access to legal abortion as a human right under certain circumstances. In General Comment No. 2 of 28 November 2014 of the African Commission on Human and Peoples’ Rights, we read that the so-called Maputo Protocol of 2003 “is the very first treaty to recognize abortion, under certain conditions, as women’s human right which they should enjoy without restriction or fear of being prosecuted” (ACHPR 2014: 2). The Maputo Protocol has been ratified by 42 countries of the African Union as of October 2019. Its Art. 14, Sec. 2 c) obliges the states parties to „protect the reproductive rights of women by authorizing medical abortion in cases of sexual assault, rape, incest, and where the continued pregnancy endangers the mental and physical health of the mother or the life of the mother or the foetus”. While the list of conditions for legal abortion included in the Maputo Protocol is nearer to the more restrictive extreme of the global scale, access to abortion under these conditions has been set forth as a human right.

In the European and Inter-American systems for the protection of human rights the regulation of abortion has not been included in the treatise order and is shaped by jurisprudence (Fewncik 2012; O’Connell 2014; Puppinck 2013).

Art. 4 Sec. 1 of the American Convention on Human Rights of 22 November 1969 stipulated that “every person has the right to have his life respected. This right shall be protected by law and, in general, from the moment of conception” (OAS 1969). While the protection of life from the moment of conception would seem to be an indirect prohibition of abortion, the jurisprudence of the Inter-American Court of Human Rights includes cases in which the Court has found abortion to be in accordance with human rights on the basis of the prohibition of torture or inhuman or degrading treatment. The recent judgment in *Manuela y otros vs. El Salvador* (2021)*,* decided by the Inter-American Court on 2 November 2021, while not going as far as the claimants had hoped for, constitutes an important development, as the Court has expressly called on El Salvador to change its regulations regarding the application of penal sanctions for infanticide, which at present also include the criminalisation of abortion (“*adecuar su regulación relativa a la dosimetría de la pena del infanticidio*”, *Manuela y otros vs. El Salvador*, Sec. 14, p. 92).^2^

The status of abortion under the European Convention on Human Rights of 4 November 1950 is determined by the jurisprudence of the European Court of Human Rights. The Court has repeatedly stressed the necessity for abortion law to “be shaped in a coherent manner which allows the different legitimate interests involved to be taken into account adequately and in accordance with the obligations deriving from the Convention” (*A, B and C v. Ireland* 2021). Probably because no right to abortion is provided for in the Convention, the “European Court of Human Rights’ record on access to abortion is largely one of avoidance”, as Bríd Ní Ghráinne and Aisling McMahon wrote in 2019 (pp. 561–584). They further argued that:

The ECtHR has tended to avoid direct encroachment on States’ discretion by finding that the respective cases do not meet the Article 3 threshold (prohibition of torture or inhuman or degrading treatment) of the European Convention on Human Rights (…). It has also held that States enjoy a wide margin of appreciation in relation to Article 8 (right to private and family life) and has only found violations of Article 8 where abortion was provided for in domestic law if it is not accessible in practice. Unsurprisingly, the dominant position in the literature is that the ECtHR is not a fruitful avenue to challenge a State’s prohibition of abortion (references of the original have been omitted) (p. 561).

Poland joined the Council of Europe in 1991, and it has been a party to the European Convention since 1993. The jurisprudence of the ECtHR in abortion-related cases against Poland provides an ample illustration for the thesis by Ní Ghráinne and McMahon. So far, Poland has lost three such cases (*R.R. v. Poland *2021, *Tysiąc v. Poland* 2021, and *P. and S. v. Poland*). All of them concerned the situation in which a pregnant woman would not be granted access to legal medical diagnostics and treatment, including abortion. In these cases, the ECtHR has found Poland to be in violation of Article 3 in various respects (the right to be free from inhuman and degrading treatment), Article 5 (the right to liberty and security) and Article 8 (the right to respect for private life) of the Convention. Since the issuing of these judgments by the ECtHR, the Polish authorities have not introduced the necessary legal changes, of which the Polish government is regularly reminded by the Committee of Ministers of the Council of Europe (Helsińska Fundacja Praw Człowieka 2021, p. 5). In January 2020, the ECtHR communicated to the Polish government a new complaint regarding the inability to have access to a legal abortion, based on Articles 3 and 8 (*B.B. v. Poland*, 2021 pending). In July 2021, the ECtHR communicated to the Polish government twelve new complaints concerning abortion rights in Poland. The Court has also notified the government of Poland that it has received “over 1000 similar applications” (ECTHR 2021, p. 1). The Council of Europe Commissioner for Human Rights has intervened in these twelve cases in October 2021, stating that:

In the Commissioner’s view, the near-total ban on abortions needs to be seen also in the wider context of the long-standing disregard for women’s sexual and reproductive health and rights in general. As noted in the Commissioner’s Rule 9 submission to the Committee of Ministers, made in January 2020, the situation in the area of sexual and reproductive health and rights in Poland has not only failed to improve, but has actually worsened in the past several years (Commissioner for Human Rights 2021, p. 4).

## A Brief History of Abortion Law in Poland After 1945

The history of abortion law in Poland from 1993 until 2020 should be read in the broader context of abortion regulation in Poland, but also of the former Eastern bloc and the region of East Central Europe (see Bunikowski 2019, p. 87; Dellapenna 2006, p. 516; Fuszara 1991). Against the backdrop of regional legal history Poland, as is so often the case, displays a number of particularities resulting first and foremost from the country’s unique cultural and especially religious landscape. If “abortion legislation in Poland’s modern history may be described as taking one step forward and two steps back”, as Julia Kapelańska-Pręgowska has recently argued (2021), this is largely a product of the intricacies of its cultural embedding.

The Communist regime established in Poland after the Second World War was—here as elsewhere—ostentatiously inimical to religion, even though the dynamic of the relations between the Communist government and the Church throughout the post-war period greatly fluctuated (e.g. Korbonski 2000; Mazgaj 2010). The Church was not “in opposition” as such, but it was the next best thing, and the largest organized supporter of the opposition whenever it emerged (Hierleman 2015, pp. 79–80). The Church and the party were constantly forcing each other’s hand, especially during the pontificate of John Paul II, whose contribution to overturning Communism is constantly debated both in politics and in academic research (e.g. Zagacki 2001; Kraszewski 2012). After 1956, when the Stalinist period marked by open state hostility to the Church ended in Poland, the Polish Communist authorities usually refrained from massive organized anti-Catholic actions, which was without prejudice to small-scale persecution, bullying, and violence, including cases such as the brutal murder of the priest Jerzy Popiełuszko in 1984. Despite the infiltration of the Church by the security service and the close monitoring of the Church’s foreign contacts it remained the only (largely) state-independent, internationally connected, large-scale social, cultural and political force in the Eastern Bloc (e.g. Kschyk 2015). Under these conditions, the regulation of abortion in the Polish People’s Republic with its almost uniformly Catholic society was a particularly telling expression of the Party’s anti-Catholic stance.

Until 1956, abortion was governed by the interwar provisions of the Criminal Code of 1932 which was reinstated after the Nazi occupation of Poland ended in 1945. The pre-war regulation was relatively liberal at the time, allowing for abortion on medical grounds and in the case of a pregnancy resulting from a crime. In 1950, a few restrictions regarding the pre-abortion documentation requirements were added by the Act on the Medical Profession. However, in 1956 the new legal regime of abortion was laid down in the Act on the Conditions for the Termination of Pregnancy which remained in force until 1993. It allowed for abortion on medical grounds, in the case of a pregnancy resulting from a crime, but also due to the ‘difficult living conditions’ of the pregnant woman (Art. 1, Sec. 1). The requirement to document the ‘difficult conditions’ was soon dropped and in fact abortion was available on request. The Catholic pro-life activists reacted with anti-abortion campaigns in churches and other public actions, such as the petition to Parliament submitted in 1977 by a relatively marginal, Church-sponsored and nationalistically-inclined oppositional Committee on Defence of Life, Family and Nation (Sikorski 2012, p. 101), signed by 12,000 citizens (Minutes from the Proceedings of the Senate Commission 2017). The goal of this action was not really to effect a legal change—hardly imaginable under the circumstances—but to increase the public awareness of the moral problems related to abortion from the Catholic perspective. At the same time, the pro-life positions at the time were insistent on the connection between abortion and national well-being. The Communist authorities were represented as enemies of the Polish nation and the legislation allowing abortion on request was seen as being detrimental to the nation’s existence in biological terms, an act of anti-Polish Communist biopolitics. Abortion was framed as a pivotal issue from the point of view of national-conservative political forces concerned for Polish national identity long before 1989.

This framing was by no means a Polish specialty: a similar connection between the construction of national identity and the approach to the legal regulation of abortion has been found in other countries, also in comparative studies showing that Catholicism is by no means particularly susceptible to producing the kind of conceptual nexus described here (Fletcher 2005; Frank and Çelik 2017; Halkias 2004; Kozlowska et al. 2016; Yeganeh 1993). Furthermore, the concern for the biological survival of the nation is also connected to considerations of the number of legally performed abortions in societies where it does not result from religious and political debates but from post-colonial cultural and demographic ones. For example, in the case of Australia it has been argued that “debates over abortion can become a site where politicians, journalists and other influential social commentators displace and assuage anxieties regarding the size and constitution of Australia's future population” (Millar 2015, p. 82). Racial, gender, cultural and national motives all play a role in the legal governance of abortion on the nation-state level.

In Poland, for important historical reasons, the national framing of abortion went hand in hand with the religious one, which provided a great potential for the universalisation of nation-centred biopolitical concerns. National conservative pro-life forces were guided by the teachings of the Catholic Church and, in particular, Paul VI’s encyclical *Humanae vitae* (Paul VI 1968). This fundamental Church document in matters of sexual ethics and procreation does not use the language of human rights. However, in the next highest-ranking milestone in the Catholic teachings on abortion, which is John Paul II’s encyclical *Evangelium vitae* (John Paul II 1995), the role of the language of human rights is very prominent: the Church’s opposition to abortion (alongside other condemned practices such as euthanasia and contraception) is framed as a consequence of its commitment to the “defence of the world's poor, those who are threatened and despised and whose human rights are violated” (John Paul II 1995, sec. 5). The encyclical stresses the right to life as a foremost human right: it speaks of “fundamental human rights, beginning with the right to life of every innocent human being” (Sec. 91). Abortion is interpreted as a violation of that right as well as of human dignity, thus to be seen as a “direct threat to the entire culture of human rights” (Sec. 18). The struggle against what would soon be called “the civilisation of death” is thus construed as a part of the global human rights agenda followed by the Catholic Church (Molek-Kozakowska 2017). This construction of the relation between human rights and abortion laws has remained, until now, as underpinning the national-conservative discourse on abortion in Poland.

The political discussion on abortion was unleashed immediately in 1989, which in itself certifies to the political energy accumulated since 1956. Even though any causal link is very difficult to establish here, it must be observed that since the 1980s, the statistics show a drop in the number of legal abortions (Bunikowski 2019, p. 100). In the early phase of the debate on abortion in democratic Poland, the catalogue of problems was established which is characteristic of the legal governance of abortion all over the world (Standish 1998). It included the scaling from the least debatable conditions to legal abortion including the danger to the life or health of the pregnant woman up to the abortion on request. The scope of criminal responsibility for an illegal abortion was another issue. Finally, the periods during which an abortion would be legal were debated, with a considerable variety of solutions being advanced.

In those days, Poland—as most post-communist states—had not yet adopted a new, post-Communist constitution, and under these circumstances the decision on abortion had a huge momentum. It was commonly and correctly believed that the way in which the problem of abortion is decided would be prognostic of the worldview landscape of democratic Polish society, and of the constitutional identity of the democratic Polish state. At stake were the role of the Catholic Church, the model of state and church relations, but also the role of the religious worldview and religious values, which were identified as the source of national identity by a wide range of political forces far beyond the conservative right (Meyer Resende 2015). All these issues would reverberate in the hectic constitutional debate of the 1990s (Blokker 2020; Hałas 2005). On 7 January 1993, the abortion law was finally adopted and it still remains in force today: the Act on Family Planning, the Protection of the Human Foetus and the Conditions for the Admissibility of Pregnancy Termination (further: “Act on Family Planning”). The regulation was henceforth commonly referred to as the ‘abortion compromise’, even though the left read it as an endorsement of the Catholic worldview, whereas the political forces close to the Church never ceased to contest it as a half-measure. Under the ‘abortion compromise’ pregnancy could not be terminated for social reasons. The pre-war catalogue of conditions in which abortion would be legal, including a danger to the woman’s life or health and a pregnancy resulting from a crime, was supplemented by one premise which entered the arena of abortion debates fairly late, with the rampant progress of medical technology: ‘grave and irreparable’ foetal impairment (Art. 7 Sec. 2). Initially, the conditions for legal abortion were formulated as exceptions to the provisions of the Criminal Code of 1969 introduced by the same Act on Family Planning, according to which causing the death of a conceived child (the wording of the Act) was subject to up to two years’ imprisonment (the mother of the conceived child being exempt). In 1996, the conditions for legal abortion were moved to Art. 4a of the Act on Family Planning. In the subsequently adopted Criminal Code of 1997, abortion was penalized by Art. 152, with up to three years’ imprisonment, and up to eight years, if the abortion would be performed on a conceived child capable of living beyond the mother’s body. The pregnant woman’s responsibility for abortion based on this article was excluded (Bojke and Wantoła 2015).

Until 1996, no party could gather enough support to be able to press for a change to the 1993 compromise in either direction. However, in 1996 the post-communist government proposed an amendment which allowed for abortion due to difficult living conditions or a difficult personal situation, and this was subsequently adopted and it came into force on 4 January 1997 (the Act on the Amendment to the Act on Family Planning 1996). The number of legal abortions rose rapidly, from 505 in 1996 to 3176 in 1997 (Bunikowski 2019, p. 100).^3^ However, a motion for a constitutional review was filed immediately and the amendment was overturned by the Constitutional Tribunal in its ruling of 8 May 1997, effective from 23 December 1997 (K 26/96). So, the last semi-successful attempt to change the abortion compromise was undertaken before the new constitutional order was determined with the entry into force, on 17 October 1997, of the Constitution of Poland of 2 April 1997. It was the Constitutional Tribunal’s ruling along with three dissenting opinions that pre-emptively sealed the link between the emergent constitutional identity of Poland and the understanding of the right to life which excluded abortion for reasons other a threat to another life (that of the pregnant woman), a crime or foetal impairment.

The standard of law enforcement in respect of the abortion compromise was far from perfect: undisputedly legal abortion was not easily available, with long procedures and doctors using the conscience clause very freely (ASTRA Network 2020, p. 82). The European Court of Human Rights has on three occasions found Poland guilty of failing to secure access to prenatal diagnostics and legal abortion (*P. and S. v. Poland*, *R.R. v. Poland, Tysiąc v. Poland*). However, Poland has never followed the Court’s recommendations regarding the human rights of pregnant women demanding a legal abortion. That said, the number of legally performed abortions has been increasing (from 124 in 2001 up to 1110 in 2019), and in 2019 over 96% thereof were due to foetal impairment, the so-called embriopathological condition. This condition for legality of abortion has been the main target of the pro-life movement since 1993. There were attempts to abolish it by the right, some of them enjoying massive popular support. In 2017, a draft bill signed by over 100,000 citizens was introduced to Parliament, proposing to remove the embriopathological condition for an abortion. The left never managed to mobilize comparable support to shift the compromise the other way. It is very probable that the ruling of the Constitutional Tribunal of 1997 played a decisive role in the relatively weaker mobilization of the advocates of the liberalisation of the abortion law: any amendment would end up in the Constitutional Tribunal, and the Tribunal tended to honour the *stare decisis*.

But it seemed that the abortion compromise was resilient enough to last even after 2015, when a declared pro-life party (Law and Justice) enjoying the Catholic Church’s support took over the state’s helm and abortion returned to the legislative agenda. In 2016, a draft law was submitted to delegalize abortion altogether. When the draft was moved to the reading stage in the parliamentary commissions, a wave of demonstrations followed in the whole country, the so-called ‘Black Protest’ (Korolczuk 2016). Law and Justice decided not to proceed with the draft law any further. Politically, it seemed a prudent course to take. In a series of surveys conducted between 1992 and 2016 by the Public Opinion Research Centre in Warsaw (CBOS 2016), support for abortion on request has shrunk from 27 to 14%, probably reflecting the intensity of the campaigning by the pro-life movement. However, the support for abortion due to a danger to the pregnant woman’s life has never decreased below 77%, and in the case of a pregnancy resulting from a crime and – in below 72%. According to the same study, the support for the legality of abortion when it is certain that a child will be mentally disabled dropped from 71% in 1992 to 53% in 2016; 30% of respondents were of the opinion that abortion should not be legal in such a case. A decisive majority of the respondents (75–76%, an increase of between 30 and 20 per cent compared to 1999) opposed the legality of abortion on the basis of a difficult material or personal situation or when a woman does not wish to have a child. This means that in 2016, the majority of Poles were not supportive of the liberalization of the abortion law. But it also means that in the same year, 53% of Poles supported the most controversial condition for the legality of abortion under the 1993 compromise, which was foetal impairment. The Black Protest seems to have had a strong influence on the acceptance of abortion. The surveys conducted after 2016 provide evidence of increasing support for liberalization as well as a growing polarization of opinions on the matter (CBOS 2020a, b: 4, 6). Crucially, however, at no time before 2020 did the comparative survey data show support for restricting the 1993 compromise to be significantly above 30%.

## The Background of the Ruling of the Constitutional Tribunal of 22 October 2020

The breakthrough in what was, politically speaking, an abortion impasse would be brought about—in 2020 just as in 1997—by the Constitutional Tribunal’s ruling. But it was a very different institution in 2020 than in 1997.

While the Constitutional Tribunal had been the target of criticism ever since its establishment in 1985, that critique usually pertained to the jurisprudential style adopted by it (e.g. Moskal 2018), as well as the resulting difficulties in the application of the Tribunal’s jurisprudence by the judiciary (Bernatt et al. 2013). The conservative position of the Tribunal in politically sensitive matters was commented upon frequently, and the aforementioned ruling on abortion of 1997 was one of those decisions which best illustrate the tendency of the Tribunal to prefer not only conservative values, but also conservative approaches in its constitutional interpretation (e.g. Waniek 2012). Last but not least, the critics would point out that the Tribunal had not developed its own position between judicial activism and passivism. Constitutional courts have been known to play prominent roles and to undertake judicial interventions, even under the conditions of a constitutional breakdown (Brown and Waller 2016). The Polish Constitutional Tribunal—whose operations also began at the moment of constitutional discontinuity between the socialist and the democratic state—has not consequently endorsed the path of activism: until 2015, its role remained somewhere in between. Nonetheless, its systemic position and its legitimation as an institution have never been systematically challenged, and it seemed that the Tribunal was indeed a functional element of the constitutional order established after 1997 (Soja 2016).

What the former constitutional judge Mirosław Wyrzykowski described as an “unconstitutional change of the constitutional system” (Wójcik 2017) had started with a conflict between the governing party and the Constitutional Tribunal in late 2015, as a result of which the Tribunal, one year later, became an enabler of governmental politics and was effectively eliminated as an instance exercising control over the system of the separation of powers (Bunikowski 2018; Łętowska 2019; Matczak 2017; Sadurski 2018, 2019; Wójcik 2017). By a series of amendments to the statutes governing the operations of the Constitutional Tribunal the Law and Justice parliamentary majority secured the support of the majority of the Tribunal’s judges, including the President of the Tribunal. The conformity of these amendments with the Constitution has been questioned by leading scholars in the country, and serious doubts have been expressed as to the binding force of the current Tribunal’s decisions (for an overview, see Sadurski 2018). However, as these doubts pertain to the highest organ of the judiciary, there is therefore no instance that is competent to review the legality of the Tribunal’s decisions. It might be viewed as a textbook case of the “*quis custodiet ipsos custodes*” paradox, but it also reveals a deep vulnerability of the constitutional system in the face of an “illiberal constitutional practice” applied by the governing majority (Uitz 2020).

The conflict with the Constitutional Tribunal was a preliminary step towards and a portent of further developments. The situation in Poland since 2015 has by now been covered extensively in the scholarship focusing on the legal, political and social components of the process (e.g. Bucholc 2019; Bucholc and Komornik 2018; Bunikowski 2018; Śledzińska-Simon 2018; Sata and Karolewski 2019). Sadurski (2019) has offered a comprehensive book-size analysis of the first phase of the process resulting in a threat to the rule of law in Poland up until 2018. Krygier (2019) has analysed the dismantling of the institutional order which had emerged after the post-communist transformation, discussing the challenge to the rule of law in Poland by right-wing populism. Since then, contributions have been made to the diagnosis of the state of the rule of law in Poland, discussing first and foremost the state of the judiciary, but also the perspectives of the public media, the private media sector, and the situation of minorities, in particular sexual minorities (e.g. Demczuk 2020; Girdwoyń 2015; Godisz and Radłuszko 2018; Surowiec et al. 2019). The scholarly findings on the decreasing standard of the rule of law in Poland have been supported by the jurisprudence of the Court of Justice of the European Union and by the communications and resolutions of the European Commission, the European Parliament, and the Council of Europe. Evidence is also provided by the reports of international organisations, including a recent report by the OSCE Office for Democratic Institutions and Human Rights on the 2020 presidential elections (ODIHR 2020), and the 2020 Memorandum on the stigmatisation of LGBTI people in Poland by the Council of Europe Commissioner for Human Rights (CMFHR 2020).

The changes to the Constitutional Tribunal have also changed the rationale for politicians to seek its decision. For the opposition it seems that bringing its cases for a review by a Tribunal composed of Law and Justice nominees is bound to be of no avail, unless this is done for purely symbolic purposes. The governing party, which controls the parliamentary majority in the lower chamber (since 2019, no longer in the upper chamber), as well as the president’s office (with a right of veto), should have little need for a constitutional review of ordinary statutes, as its legislative goals may be achieved without recourse thereto. However, in some cases it may be politically advisable to accomplish a legal change by way of constitutional jurisprudence instead of the usual course of parliamentary lawmaking. This is, essentially, what happened in the case of the abortion law.

The motion for a constitutional review of Art. 4a (Sec. 1, 2) of the Act on Family Planning (foetal impairment) had first been submitted in October 2017 by a group of right-wing MPs (K 13/17). One of the signatories of the motion was subsequently, in December 2019, nominated to the Constitutional Tribunal. The same person and another of the current judges of the Constitutional Tribunal have been Members of Parliament in the two terms between 2011 and 2019, and both of them participated in the parliamentary works regarding the opinion on motion K 13/17. As judges of the Tribunal, they would thereby rule on a matter in which they were previously involved as members of the lawmaking body. In the same year, 2017, on the 30th of November, the aforementioned citizens’ draft bill for an amendment to the Act on Family Planning was submitted to the lower chamber of Parliament which has gone through one reading in the Sejm. The parliamentary works were formally continued after the 2019 elections, but they were effectively halted indefinitely. The 2017 motion to the Constitutional Tribunal was also frozen. It became something of a sword of Damocles: after the end of the parliamentary term in 2019, it had then been discontinued, only to be re-submitted in substantially the same form immediately after the parliamentary elections.

The sword fell in the autumn of 2020. The ruling of 22 October 2020 (K 1/20) declared that Art. 4a Sec. 1, 2) of the Act on Family Planning (abortion for foetal impairment) was unconstitutional. The ruling was decided by the Tribunal *in corpore*, with five dissenting opinions.^4^ Upon the announcement of the ruling an unprecedented wave of protests spread through the country, with hundreds of thousands of protesters on the streets despite the restrictions related to the COVID-19 pandemic (Bucholc and Komornik 2020a, b). The protests were noted not only in the biggest cities, but also in small towns with little tradition of civic engagement. Women’s movements and organizations resorted to unprecedentedly strong language and radical rhetoric that thus far had been unusual in Polish political demonstrations. The diagnosis of a cultural war was accepted by both sides to the conflict, and the most fundamentalist advocates of the pro-life and pro-choice positions became the most vocal in public. According to the survey results published in December 2020, 63% of Polish society supported the protesters, even though only 8% declared that they had personally participated in the protests (CBOS 2020a, b, p. 10). The lines of political radicalisation revived and reasserted the nationalist framing of the abortion debate, and they also brought about an unprecedented intensification of anti-clerical attitudes and actions, including interruptions to religious services. On 27 October 2020, the leader of Law and Justice, Jarosław Kaczyński, publicly called upon Poles to ‘take to the streets to defend our churches’ and to prevent the destruction of Poland. Various nationalist and extremist groups readily responded.

During this hectic period, numerous legal experts challenged the ruling. Formal objections as to the legal capacity of the judges sitting on the panel were invoked, owing either to misgivings concerning their nomination process or to their previous engagement in preparing and submitting the motion to the Constitutional Tribunal (Łętowska 2020). Formal issues regarding a violation of the *nemo iudex in causa sua* principle were summarized in the dissenting opinion by Judge Leon Kieres (K 1/20, Sec. 2.2). Many further issues were raised, two among which seem particularly important.

The first substantive point concerned the practice of using the Constitutional Tribunal as an alternative legislator contrary to its constitutional position as a specialized part of the judiciary. Since at the time of judging there was a plan to amend the Act on Family Planning being proceeded by Parliament, no legal reason existed to seek a constitutional review of a law which was about to be amended just before the legislative procedure closed. It was argued that the inaction of the legislature in respect of the draft amendment of the Act should not be used to instrumentalize the Tribunal to step in for the lawmaker. For this reason, according to the dissenting opinion by Judge Kieres, the Tribunal should have rejected the motion as being formally inadmissible (K 1/20, Sec. 1.3, 3.3.2, 3.5.1–3.5.2).

The second point was related to the fact that as a consequence of the Tribunal’s ruling the exception regarding the criminal responsibility for abortion vanished from the legal system. The result is an extension of the criminal sanction to cases of abortion which were not previously crimes—the creation of a new type of crime. This is contrary to Art. 42 Sec. 1 of the Constitution, according to which a crime should be defined by a statute. In the ruling of October 2020, the Constitutional Tribunal not only assumed the role of an alternative legislator, it created an entirely new criminal norm (see the dissenting opinions of Judges Piotr Pszczółkowski, K 1/20, Sec. 6, and Leon Kieres, K 1/20, Sec. 3.4.3).

It is noteworthy that while many voices were raised both for and against the ruling and the new situation which it created, the ruling itself had not yet entered into force—it was still awaiting publication. The publication of the Tribunal’s rulings is the task of the government, and the government was delaying it; Law and Justice had done the same in 2015 (Szuleka et al. 2016: 21). While there is no conceivable legal justification for the delay, the political justification seemed to be the need to await social reactions. A state of legal uncertainty was thus created: hospitals were reported to be acting as though the ruling was already in force, thereby cancelling scheduled legal abortions (Chrzczonowicz 2020). Nonetheless, despite the wave of unceasing if wavering protests, the ruling was finally published and it entered into force on 27 January 2021.

## Human Rights in the Constitutional Tribunal’s Ruling on Abortion Law of 22 October 2020

In my analysis of the Constitutional Tribunal’s abortion ruling of 22 October 2020 I do not consider the legality of this ruling. This should not be read as a commitment of any kind—my reasons are purely methodological. First, even if the ruling were non-existent or invalid, there is no mechanism for controlling the validity of the Tribunal’s decisions. Therefore, an invalid or a non-existent ruling will nonetheless be effective as long as it is enforced. The ruling of 22 October 2020 has been published and is being applied in the practice of the healthcare system. Second, as there is no legal method to eliminate the said ruling from the legal system, it will remain as a part thereof and as a possible reference. While it is conceivable that the jurisprudence of the Tribunal from a certain period will simply be discarded in later practice and remain a relic of the systemic irregularity from the era of democratic backsliding, it is equally imaginable that the ruling may form the basis for the new and stable jurisprudential practice.

The legal reasoning contained in the ruling (K 1/20, Part III) is very concise (22 pages), the remainder of the 153 pages of the published version comprising five dissenting opinions. In this section, I discuss the Tribunal’s motives as indicating either a broadening or a closing of the jurisprudential horizon.

### The Liberal Eugenic and the Definition of a Human Being: Opening Jurisprudence to Politics

The Tribunal, following the previous jurisprudence, stressed its adherence to the understanding of the legal system as a “cultural creation, embedded in the historical experience of the community and construed based on the system of values common to a given circle of subjects” (K 44/07, cited in: K 1/20, Sec. 1.2). It bespeaks the cultural embedding of the Tribunal’s ruling that there are exactly two moments in its reasoning which expressly broaden its jurisprudential horizon by opening it to politics.

The first such moment concerns the concept of “liberal eugenics”. In the motion for constitutional review in the case, which was submitted by a group of MPs, a question was posed as to whether Art. 4 Sec. 1 Item 2 of the Act on Family Planning “legalizes eugenic practices” in respect of unborn children. The Tribunal did not question the constitutional relevance of this question. Instead, it proceeded to analyse the norm in question as allowing for a “eugenic practice” (K 1/20, Sec. 2.3).

To decide whether or not the norm under consideration had a eugenic character, the Tribunal cited a range of contemporary philosophers, including Francis Fukuyama, Michael Sandel, and Jürgen Habermas. This openness to non-juristic arguments and interpretations of social reality would in itself be a commendable effort to offer a deeper insight into the matter at hand. This is not so rare in the jurisprudence of some constitutional courts, notably the US Supreme Court, which has come to include not only traditional philosophical and religious arguments, but also social-scientific, psychological and economic ones in its reasoning (Petersen 2013). The consideration of philosophical arguments led the Tribunal to opine that the admissibility of abortion due to foetal impairment “has a eugenic character in the sense of liberal eugenics” (K 1/20, Sec. 2.3 *in fine*). Thus, the Tribunal, following the wording of the motion for a constitutional review, responded to the question of the motion by pronouncing a qualification of a legal norm in extra-legal, philosophical categories whose considerable rhetorical potential derives from the negative connotation of the term “eugenics”, but which have no direct constitutional bearing (this point was raised in Judge Kieres’ dissenting opinion, Sec. 5.4.2). Thus, the ruling brings to the Polish constitutional jurisprudence on abortion an opening of the jurisprudential horizon towards the political critique of liberalism. The decision on the eugenic character of the analysed norm has framed the whole argumentation of the Tribunal as a legal specification of liberal eugenics. It should be noted that the long passage on the eugenic character of the norm under consideration seems at odds with the following concluding statement (K 1/20 Sec. 3.4):

The Tribunal notes that it does not refer, as it did not refer earlier, to arguments of a philosophical nature, or more precisely, to ethical arguments relating to the status of man and the beginning of human life. The Tribunal bases its conclusions on purely normative premises resulting from the belief that the legal system is coherent, in particular axiologically (…).

The Tribunal did not see the presumption of the value coherence of the legal system as a reason to refrain from seeking a philosophical qualification of a legal norm. This implies the legal relevance of the value positions from which such a philosophical qualification stems. This opening of the jurisprudential horizon to references from beyond the legal system allows for an extra-constitutional and extra-legal assessment of a legal norm. It is a significant move, because this opening came about following a motion by politicians: The Tribunal chose not to draw a line to demarcate the political system from the legal one. Instead, it followed the qualification made within the political system and transposed it directly into the legal system, making the concept of liberal eugenics a part of constitutional jurisprudence.

The second opening to politics originated from the discussion of the concept of a human (K 1/20, Sec. 3.3.3). On this point, the Tribunal, in order to decide whether a foetus or an unborn child can be considered to be human for the purposes of constitutional review, decided to refer to the files of the Constitutional Commission of the National Assembly, which was the body responsible for drafting the Constitution of 1997. It is by no means uncommon in the Constitutional Tribunal’s jurisprudence to refer to parliamentary files (K 29/96, Sec. 3 and the dissenting opinion of Judge Czeszejko-Sochacki on this point). The files are used to supplement legal reasoning and to support a historical and so-called authentic interpretation (establishing the original meaning of the norm as conceived by the constitutional lawmaker). The detailed and extensive discussion of the files of the Constitutional Commission in the Tribunal’s ruling of 2020 is an uncharacteristically originalist turn in Polish constitutional jurisprudence. It may also be considered to represent a tendency that is contrary to the opening to philosophical references discussed above: while the philosophical ideas tend to consider humanity *sub specie aeternitatis*, the discussion in the Constitutional Commission was a historically situated political debate. Nonetheless, what deserves the most attention is the goal for which the files were used. Based on their interpretation, the Tribunal construed an argument according to which it was the intention of the Commission—and thus of the constitutional lawmaker in 1997—to leave defining the human being in the context of abortion law to the Constitutional Tribunal. The goal of the historical interpretation in this case was, therefore, not to support the understanding of the legal norm which remained unclear (as would be customary in the Polish legal culture), but to define the sphere of competence of the Tribunal in the system of the separation of state powers. As a consequence, the files of the Constitutional Commission were used as political legitimation to expand the right of the Tribunal at the expense of legislative authority.

### Human Dignity, Life and Motherhood: Closing Jurisprudence to International Interdependence

The Tribunal took human dignity as a starting point in its reasoning, stressing the priority of human dignity in respect of the constitutional system (the “source of liberties and rights”, Art. 30 of the Constitution) and defining dignity as a “certain kind of an opening of the constitutional system to extra-legal and pre-legal values” (K 1/20, Sec. 3.1). However, the Tribunal did not decide to use any further extra-legal sources as references to support its reasoning in this respect.^5^ In a next step, it pointed out that the right to life is a consequence of human dignity, and, citing previous jurisprudence, made anew a connection between the democratic rule of law and the protection of dignity and life: “The Tribunal has repeatedly emphasized that’a democratic state ruled by law is a state based on respect for human beings, and in particular on respect for and the protection of life and human dignity. These two goods are directly related.’” (K 1/20, Sec. 3.2).

The selective continuity in the jurisprudential practice of the Tribunal since the 1990s is evident in the analysed ruling, as is the pivotal importance of the Tribunal’s ruling which overturned the liberalization of the abortion law in 1997 (K 26/96).^6^ The latter decision is cited 16 times over 35 pages, and almost the whole of Sec. 3.3.1. of the 2020 ruling literally repeats the motives in the 1997 decision. But references to international law, including the International Covenant on Civil and Political Rights, the European Convention for the Protection of Human Rights and Fundamental Freedoms, and the Charter of Fundamental Rights of the European Union, are limited to the interpretation of the applicability of the protection of the right to life to the life of a human being in the prenatal period, and beyond that context they are only found in quotations. No reference was made to the jurisprudence of international courts.^7^

The consideration of human dignity and the right to life in the Tribunal’s ruling does not include any reference to the dignity of a woman, despite the fact that the problem of the relationship between the rights of the pregnant woman and the rights of the human being in the prenatal phase of its life had been raised in the motion for constitutional review and by the *amici curiae*. In his dissenting opinion, Judge Pszczółkowski thus commented on this lacuna in the Tribunal’s reasoning:

The Constitutional Tribunal (…) only considered one perspective—the perspective of protecting life in the prenatal phase. At the same time, it ignored the perspective of women whose dignity, life and health are undoubtedly values under constitutional protection. In the name of protecting life in the prenatal phase—which is not absolute—the Constitutional Tribunal imposed on women the obligation to have a heroic attitude, i.e. the obligation to assume responsibility in all circumstances—regardless of the nature and degree of pathology of the foetal development or the possible consequences for their life and health with the continuation of pregnancy—for sacrifices and hardships far exceeding the usual measure of limitations related to pregnancy, childbirth and raising a child.

Similarly, respect for human beings, to which the Tribunal refers as a foundation of the rule of law (K 1/20, Sec. 3.2), only refers to one category of subjects affected by an abortion, and these subjects are defined as human beings in the prenatal phases of their lives. The interpretation of the rule of law does not consider respect for women as human beings as a value which should be weighed against other protected constitutional goods and values, with one important exception: their legal capacity as mothers.

The core of the reasoning of the Tribunal in respect of the role of the woman is a reprise of the considerations in the abortion act of 1997:

(…) [the] protection of motherhood cannot only mean the protection of the interests of a pregnant woman and mother. The use of a noun by constitutional provisions indicates a specific relationship between a woman and a child, including a child who has just been conceived. The entire relationship (…) has the character of a constitutional value, thus encompassing the life of the foetus, without which the maternity relationship would be interrupted. Therefore, the protection of motherhood cannot be understood as protection realized only from the point of view of the interests of the mother/pregnant woman (K 26/96, Sec. 3, cited in: K 1/20, Sec 3.3.1).

One semantic shift in this passage deserves particular attention. The Tribunal’s ruling of 1997 precluded the understanding of the constitutional protection of the mother and motherhood as only pertaining to the legal sphere of the pregnant woman and the mother herself. It posited motherhood and being a mother as a multilateral, relational quality, realized in the constitutionally protected family whose “basic function is procreation” (K 29/96, Sec. 3). The 2020 ruling develops this reasoning to conclude that the constitutional protection of motherhood does not allow a consideration of any rights of the pregnant woman to be effectively weighed against the protection of human life in the prenatal phase and motherhood as a relation existing from the moment of conception. In Sec. 4.2, we read: “Due to the essence of the termination of pregnancy, considering the conflict situation, an analogous good can only be sought on the side of the child's mother.” (K 1/20). The following analysis makes it clear that the analogous good to be pondered against the right to life of the unborn child is the mother’s life, nothing less:

Although a high probability of a severe and irreversible impairment of the foetus or an incurable life-threatening disease may also be associated with a threat to the life or health of the mother, Art. 4a Sec. 1, Item 2 of the Act does not apply to such a situation, the more so as the fact of such a conflict was indicated as a separate premise for the termination of pregnancy in Art. 4a Sec. 1 Item 1 of the Act.

While the 1997 ruling complicated the jurisprudential horizon in matters related to abortion by introducing the motive of the social complexity of family relations, the 2020 ruling narrows the question of abortion down to the matter of life and dilutes the whole complexity of the woman’s rights involved therein.

In the conclusion of the ruling (K 1/20, Sec. 4.2) we find the following passage:

In the opinion of the Tribunal, Art. 4a Sec. 1 Item 2 of the Act does not allow one to assume that the high probability of a severe and irreversible foetal impairment or an incurable life-threatening disease is to constitute a basis for the automatic presumption of a violation of the well-being of a pregnant woman, and the mere indication of a child's potential burden concerning such defects is eugenic in nature. This provision lacks any reference to measurable criteria of violating the mother's welfare justifying the termination of pregnancy, i.e. a situation in which she could not be legally required to sacrifice her legal interest.

While it is undoubtedly true that neither the Constitution nor the Act on Family Planning contain any precise indication as to the measurable impact of foetal impairment on the well-being of the pregnant woman, indications to that effect could easily be found in the jurisprudence of international courts and in the interpretations of the acts of international law to which Poland is a party. For example, *Mellet v. Ireland* (2021) has provided an interpretation of arbitrary or unlawful interference with a pregnant woman’s privacy and family, and it includes tools for assessing “the balance that the State party has chosen to strike between protection of the foetus and the rights of the woman” (*Mellet v. Ireland* 2021, Sec. 7.8). Similar directions for the interpretation of the conflict of constitutional goods and values could be found in the cases against Poland decided by the European Court of Human Rights (*R.R. v. Poland* 2021, *Tysiąc v. Poland* 2021, and *P. and S. v. Poland* 2021). It is a reference that is bound to emerge wherever the conflict of legal goods is considered, but it did not do so in this case (see the dissenting opinion by Judge Kieres, K 1/20, Sec. 5.7.1–5.7.4). Thus, the passage quoted above is a particularly striking moment of closing the jurisprudential horizon: it is a refusal to treat international law and jurisprudence as a reference in the interpretation of Polish law despite the fact that the Tribunal acknowledged the absence of sufficient interpretation guidance in the domestic legal system. The Tribunal did not reject or refute the findings of the international bodies: it considered them irrelevant.

## Conclusion

The public standing and the legitimacy of the Constitutional Tribunal as an institution has suffered greatly since 2015. The image of the Tribunal has never been as poor as it is now. According to the survey study by the Centre for Public Opinion Research, 50% of respondents had a negative opinion about the Tribunal’s work in December 2020, more than double the number of those who had a good opinion thereon (24%) (CBOS 2020a, b, p. 5). This is a steep decline since the 34% of favourable opinions in late 2019, not to mention the 42% of favourable and 12% of negative opinions in March 2015, before the conflict surrounding the Tribunal had emerged. This low public standing undermines any decision that the Tribunal makes, including the abortion ruling. But the Tribunal’s decision, once issued and published, affects not only social life, but also the legal system.

The reasoning of the Tribunal in the ruling of 22 October 2020 represents a twofold shift. On the one hand, there is an opening of the Tribunal’s reasoning to the intersection with politics. This is demonstrated in its adherence to the wording of the motion for a constitutional review of the Act on Family Planning as a legalisation of eugenic practices, and in the use of the files of the Constitutional Commission as a decisive argument on the Tribunal’s sphere of competence. This opening of the jurisprudential horizon to politics is accompanied by a closing of the international interdependencies of Poland as a member of the European Union, the Council of Europe and the United Nations insofar as the commitment to protect human rights according to international standards is concerned. The relevance of Poland belonging to what could be described as a global (or regional) legal culture of human rights (Arat 2006) is thus significantly decreased.

The case of abortion law in Poland confirms the point made in 2019 by Martin Krygier (545–546):

So successful were post-communist transitions presumed to be (or perhaps more soberly, nudged to become), that already by 2004, ten countries were certified (implausibly and perhaps prematurely) as having shown that they had 'achieved stability of institutions guaranteeing democracy, the rule of law, human rights, and respect for and protection of minorities', and so could be welcomed into the European Union. Now it seems that, in a parody of Groucho Marx, some of them seem reluctant to be part of a club that would have them as members. For things look different now.

Things do, indeed, look different now: the closing of the jurisprudential horizon by neglecting and disregarding the international legal order raises concerns for the protection of human rights in Poland according to international standards, including the rights of pregnant women to obtain access to a legal abortion as confirmed by international jurisprudence. But it also raises a deep concern for the rule of law. The rule of law as a constitutional principle does not prescribe any particular content of abortion law, as it is not, in the Council of Europe system, a treaty-based human right at the time of writing. But it does entail respect for international commitments and obligations. In the global world, where both the legal order and legal consciousness encompass acts of international law, legal certainty which belongs to the core meaning of the rule of law in the European Union includes a consistent application of the international standards for the protection of fundamental rights, including effective judicial review (see European Commission 2014, Sec. 2). The closing of the jurisprudential horizon means that a judicial review in matters of abortion and, by extension, other human rights matters will be limited.

Philosophically speaking, adherence to international standards is an expression of the self-limitation of the state, which makes the rule of law at all possible. A state’s international interdependencies are asserted, among other things, in the fact that the jurisprudential practice of the courts of this state includes references to international law and international jurisprudence. Disregarding international interdependencies proclaims the self-containment and legal self-reliance of a nation state, and at the same time it denies the nature of the rule of law as a common achievement of the international community. A non-obvious but direct consequence of the ruling of the Polish Constitutional Tribunal of 22 October 2020 is removing the rule of law from the international level, where the rule of law and human rights are intrinsically connected, to the national level, where they may easily be separated.

## Footnotes

1 Among the many criticisms of the concept, it should be mentioned that it has been officially used almost exclusively in respect of women’s rights and health (on men’s reproductive rights, see Totz 1994).

2 This part of the sentence was voted upon unanimously, even though there were four dissenting and concurring individual opinions in the Court’s decision, available here: https://www.corteidh.or.cr/jurisprudencia-search.cfm?lang=es&id=13.

3 Slightly lower numbers are provided in: Odpowiedź na interpelację no. 5624 [Answer to Interpellation] (2016).

4 Two of the dissenters opposed the verdict and three concurred with it, but opposing the grounds for reaching such a verdict.

5 The dissenting opinions, especially by Judge Kieres, pointed out the selective and far from exhaustive use made by the Tribunal of the scholarly literature on matters that were relevant for the ruling.

6 For a very interesting and original discussion regarding the significant discontinuity in the jurisprudential line as far as the interpretation of the constitutional term “everyone” is concerned, see Bunikowski 2021.

7 In one case, a previous judgment of the Tribunal was cited in which a general reference to the jurisprudence of the European Court of Human Rights had been made, K 1/20, Sec. 4.2.
